# Endocardite de Libman-Sacks surinfectée: à propos d'un cas

**DOI:** 10.11604/pamj.2019.33.97.9597

**Published:** 2019-06-10

**Authors:** Lahatriniavo Ritchy Ramiandrisoa, Haingo Freddie Richard Raveloson, Daniella Masinarivo Rakotoniaina, Nirina Rabearivony, Solofonirina Rakotoarimanana

**Affiliations:** 1 Faculté de Médecine, Université d'Antananarivo, Service des Soins Intensifs Cardiologiques, CHU Befelatanana, Antananarivo, Madagascar; 2 Faculté de Médecine, Université d'Antananarivo, Service de Cardiologie, CHU Befelatanana, Antananarivo, Madagascar

**Keywords:** Endocardite de Libman-Sacks, endocardite infectieuse, lupus érythémateux disséminé, Libman-Sacks endocarditis, infective endocarditis, systemic lupus erythematosus

## Abstract

L'endocardite de Libman-Sacks constitue une manifestation cardiaque peu fréquente de la maladie lupique au cours de laquelle il existe une végétation non infectieuse au niveau des valves cardiaques. Il y a un risque important d'endocardite infectieuse. Notre patiente était une femme de 38 ans, présentant une fièvre persistante et une polyarthralgie inflammatoire au niveau des poignets et des chevilles. Elle présentait un souffle systolique mitral 2 sur 6 et une tuméfaction douloureuse des poignets et des chevilles. Nous avions objectivé un syndrome inflammatoire biologique. Le dosage des anticorps anti-nucléaire était positif avec un aspect moucheté, ainsi que les anticorps anti-DNA natifs. La recherche de syndrome des anti-phospholipides (SAPL) était négative. L'échographie doppler cardiaque avait objectivé des végétations au niveau des valves mitrales et aortiques. Les améliorations clinique, biologique et morphologique n'avaient été obtenues qu'après association antibiothérapie et corticothérapie. Nous pouvons conclure que l'endocardite de Libman-Sacks est d'évolution favorable en l'absence d'un syndrome des anti-phospholipides associé. Il faut toujours craindre dans tous les cas une greffe bactérienne. Le traitement est basé sur l'association antibiothérapie-corticothérapie-antipaludéen de synthèse.

## Abstract (en)

Libman-Sacks endocarditis is a rare cardiac manifestation systemic lupus erythematosus, in which there is a sterile vegetation in the heart valves. There is a significant risk of infective endocarditis. Our patient was a 38 year old woman with persistent fever from two months with inflammatory polyarthralgia, fixed at the wrists and ankles. She was febrile at 39 ° C, had a mitral systolic murmur 2/6 and painful swelling of the wrists and ankles. We have objectified an inflammatory syndrome, blood cultures were negative. The dosage of anti-nuclear antibody was positive with a mottled appearance, as well as anti-DNA antibodies. The Doppler echocardiography had objectified vegetations in the mitral and aortic valves. Clinical, biological and morphological improvements were obtained after antibiotic and corticosteroid combination. We can conclude that Libman-Sacks endocarditis evolution is favorable in the absence of an associated antiphospholipid syndrome (APS). Always fear in all cases a surinfection. The treatment is based on the combination antibiotic-corticosteroid-synthetic antimalarial.

## Introduction

L'endocardite de Libman-Sacks est définie par l'existence de végétations non infectieuses localisées surtout au niveau de la valve mitrale et de la valve aortique. La hantise, c'est la surinfection mais aussi la survenue dans un contexte de syndrome des anti-phospholipides qu'il faut toujours rechercher. Notre objectif était de rapporter une observation sur une endocardite de Libman-Sacks surinfectée.

## Patient et observation

**Figure 1:**
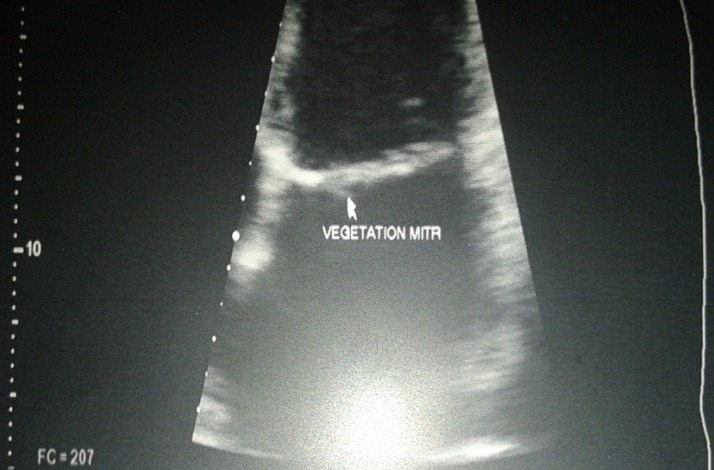
la végétation mitrale avant le traitement

**Figure 2:**
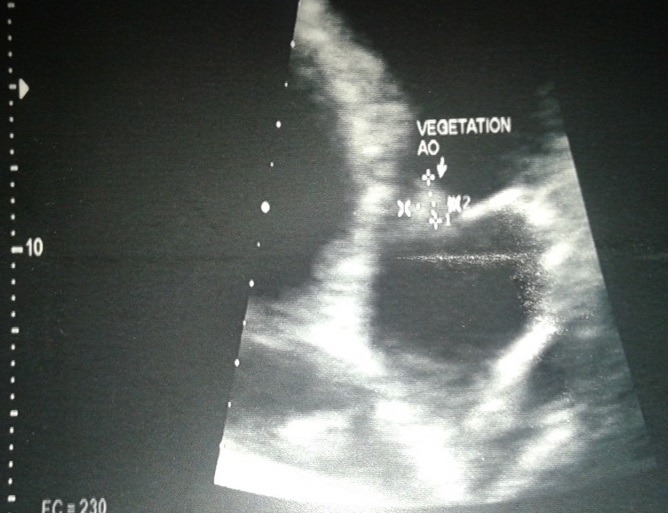
la végétation aortique avant le traitement

**Figure 3:**
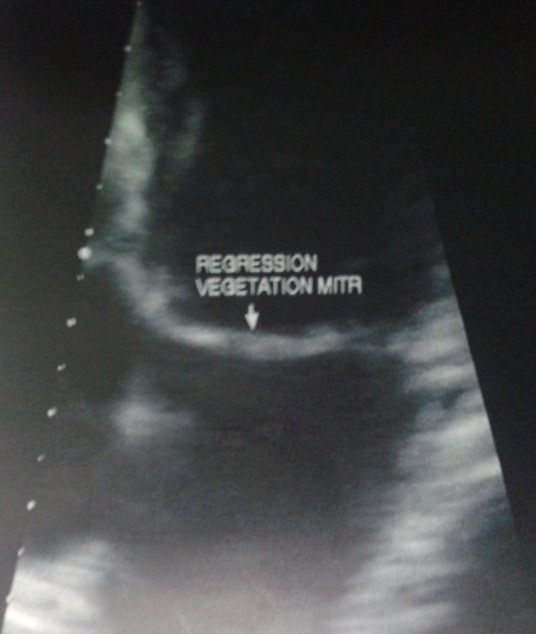
régression de la végétation mitrale

**Figure 4:**
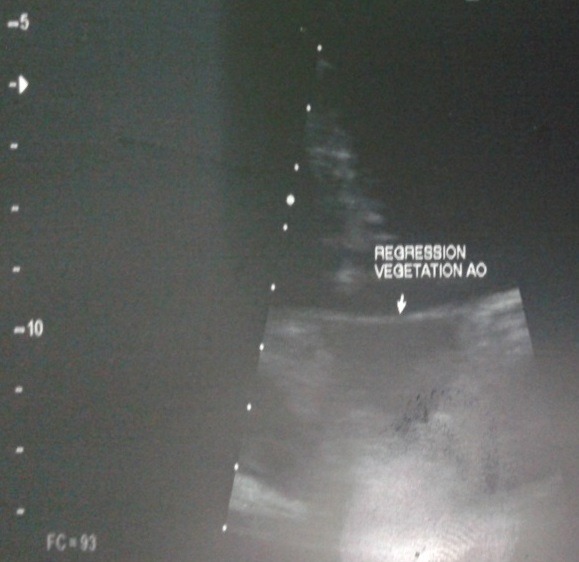
régression de la végétation aortique

La patiente était une femme de 38 ans, enseignante, qui présentait une fièvre persistante évoluant depuis deux mois, associée à une polyarthralgie d'allure inflammatoire, fixe au niveau des deux poignets et des deux chevilles. Elle n'avait pas d'antécédent particulier, pas de foyer infectieux chronique, pas de cardiopathie connue. À l'admission, elle était fébrile à 39°C. L'état hémodynamique était correct. L'auscultation cardiaque révélait un souffle systolique maximal au foyer mitral, d'intensité 2 sur 6. Par ailleurs, les deux poignets et les deux chevilles étaient tuméfiés et douloureux. À noter qu'il n'y avait pas eu de lésions cutanées évocatrices de lupus. Biologiquement, nous avions objectivé un syndrome inflammatoire avec une polynucléose neutrophile à 11 Giga par litre dont 90% de neutrophiles, un CRP élevé à 63 mg/l. Le bilan rénal était normal et les hémocultures étaient négatives, à préciser que la patiente était déjà sous antibiothérapie à l'admission. Morphologiquement, l'échographie doppler cardiaque avait objectivée des valves mitrales et aortiques remaniées avec des végétations de 5,36 mm de diamètre au niveau mitral et de 7,40 x 5,45 mm au niveau aortique (Figure 1, Figure 2). Le diagnostic d'endocardite infectieuse était alors évoqué, mais malgré une antibiothérapie bien menée, les douleurs articulaires, la fièvre et le syndrome inflammatoire biologique avaient persisté aux septièmes jours du traitement. Un bilan immunologique était alors réalisé qui avait montré un titre élevé des anticorps anti-nucléaire à 1280 UI par ml, avec un aspect moucheté. De même, les anticorps anti-ADN natifs étaient aussi positifs à 124 UI par ml. Sur ce fait, la mesure du temps de céphalines activé et le dosage du TPHA-VDRL avaient été effectué pour rechercher un éventuel SAPL associé, mais qui étaient négatifs. Une corticothérapie avait été débutée, associée à un antipaludéen de synthèse, qui avait permis une amélioration de l'état général avec disparition des douleurs articulaires, une nette régression des végétations valvulaires, plus ou moins précoce, au dixième jour d'antibiothérapie et au quatrième jour de corticothérapie (Figure 3, Figure 4).

## Discussion

L'endocardite de Libman-Sacks (autrement connu comme endocardite non bactérienne) constitue une manifestation cardiaque caractéristique du lupus systémique. Elle touche surtout les valves mitrales et aortiques mais toutes les quatre valves et toute la surface de l'endocarde peuvent cependant être touchées [1]. Les végétations sont de petites tailles et passent souvent inaperçues à l'échographie transthoracique, mais des végétations plus importantes peuvent se voir comme dans notre observation [2,3]. Roldan *et al.* ont trouvé une prévalence de 43% d'endocardite de Libman-Sacks dans une série de 69 patients lupiques par la réalisation systématique d'une échographie transoesophagienne [4]. Pour d'autres auteurs, l'endocardite de Libman-Sacks représente moins de 10% des patients atteints de lupus. Cette différence de prévalence s'explique probablement par l'absence de réalisation systématique d'échographie cardiaque transoesophagienne. La présence d'un syndrome des antiphospholipides serait un facteur favorisant la formation de végétations, mais parfois non retrouvé comme dans notre cas [2,3,5-8]. L'examen anatomopathologique des végétations montre des dépôts de fibrine, un infiltrat de cellules inflammatoires mononuclées, de la fibrose, des néovaisseaux et parfois des dépôts d'immunoglobulines et de complément [9,10]. Un diagnostic différentiel entre endocardite de Libman-Sacks et endocardite infectieuse est obligatoire. Dans cet aspect, trois données de laboratoires sont importants: numération leucocytaire, taux de CRP et les cultures de sang. Les leucocytes ont tendance à diminuer au cours de l'activité lupique, ce qui n'est pas le cas dans l'endocardite infectieuse. Un taux de CRP élevé suggère une cause infectieuse, comme les patients lupiques sont moins capables de présenter une réponse exubérante de cette protéine. Cependant, pour trancher le diagnostic, les hémocultures sont primordiales [1]. Concernant notre cas, nous avions objectivé une polynucléose neutrophile, un CRP élevé mais les hémocultures n'ont pas été concluantes, liées probablement à l'antibiothérapie reçue préalablement.

## Conclusion

Lorsque le syndrome inflammatoire clinique et biologique persiste sous antibiothérapie au cours d'une endocardite infectieuse, nous proposons alors de compléter le bilan par la recherche des anticorps antinucléaires et anti-DNA natifs, puis d'un syndrome des antiphospholipides éventuellement associé. Dans tous les cas, au cours d'une endocardite de Libman-Sacks, il faut toujours craindre une surinfection bactérienne. Le traitement sera donc basé sur l'association: antibiotiques, corticoïdes et antipaludéens de synthèse.

## Conflits d’intérêts

Les auteurs ne déclarent aucun conflit d'intérêts.
